# IN THEIR OWN WORDS: EXPERIENCES OF LONELINESS, ISOLATION, AND SERVICE UTILIZATION AMONG LGBTQ+ OLDER ADULTS

**DOI:** 10.1093/geroni/igae098.1207

**Published:** 2024-12-31

**Authors:** Christine Happel, Holly Dabelko-Schoeny, Marisa Sheldon, Al Cho

**Affiliations:** Age-Friendly Innovation Center, The Ohio State University, College of Social Work, Columbus, Ohio, United States; The Ohio State University, Columbus, Ohio, United States; The Ohio State University, Age-Friendly Innovation Center, Columbus, Ohio, United States; Age-Friendly Innovation Center, Columbus, Ohio, United States

## Abstract

Research has dismantled the stereotype of older LGBT people as lonely, depressed, and unattached to significant relationships. Yet, social isolation and loneliness are increasingly common experiences, especially when compounded by health and economic disparities. These experiences have substantial implications for our health and longevity. This study aimed to gain a better understanding of LGBTQ+ older adults’ experiences of loneliness and isolation and where and how they are connecting to community and social services to inform strategies rooted in community strengths. Through community partnerships in one Midwestern US city, semi-structured interviews were completed with 12 LGBTQ+ older adults and three service providers supporting LGBTQ+ populations. 58% of older adult participants were between 65-69. 16% were trans, and 16% were gender non-conforming. 41% were Black / African American. 92% were gay or lesbian, and 8% were bisexual. 83% had college degrees. 41% were married or living with a significant other. Interviews were analyzed using the team-based flexible coding method. Results indicated that being partnered does not necessarily relieve experiences of loneliness, and coming out later in life was associated with smaller, less satisfying support networks. Health challenges acted as both a source of positive and negative impact, as participants discussed connection to others experiencing similar challenges through support groups and social networks but also recognized limitations created by chronic conditions. Limited access to inclusive gathering spaces beyond bars, as well as the evolving queer community, were identified barriers to social connection. Intergenerational relationships were referenced as supportive and protective factors.

